# Acute Respiratory Distress in Patient with Laryngeal Schwannoma

**DOI:** 10.1155/2012/616913

**Published:** 2012-05-31

**Authors:** Laura Mannarini, Patrizia Morbini, Giulia Bertino, Omar Gatti, Marco Benazzo

**Affiliations:** ^1^Department of Otorhinolaryngology, University of Pavia, IRCCS Policlinico S. Matteo Foundation, Pavia, Italy; ^2^Post Graduation Program of Oncology, Faculty of Medicine, University of Sao Paulo (USP), 01246-000 São Paulo, SP, Brazil; ^3^Service of Pathology, University of Pavia, IRCCS Policlinico S. Matteo Foundation, Pavia, Italy

## Abstract

Schwannoma is a neurogenic benign tumour arising from the proliferation of Schwann cells present in the peripheral nerve sheath of myelinated nerves. This proliferation can hypothetically appear in every anatomic region of the human body, but the nerve sheath tumors rarely occur within the larynx. In this paper the authors discuss the case of a 74-year-old female who presented to Emergency Unit (EU) for an important acute respiratory distress. Airway flexible endoscopy revealed a bulky mass of the aryepiglottic fold measuring 3.5 cm in diameter. The patient underwent tracheotomy and a single-step surgical excision treatment of the mass which was recognized as a schwannoma at pathological examination. Tracheotomy was closed 2 weeks postoperatively. After 18 months of followup, the patient is alive and free of disease and her voice had improved markedly.

## 1. Introduction

Schwannomas were first described in 1908 by Verocay, who termed them neurinomas. In 1940, Stout recognized the schwannian derivations of this neurogenic tumors and called the same entity schwannoma. Benign neurogenic tumors include neurofibromas and schwannomas [1]. Some schwannomas have been associated with Von Recklinghausen's disease (VRD) [2]. Schwannomas are slow-growing benign, encapsulated, submucosal tumors derived from neural sheath of the Schwann's cells and occur at any age with an increased incidence in sixth and seventh decades of life, predominantly in women [3]. This tumor is very uncommon in the pediatric population [4]. Schwannomas are generally solitary and can originate in any somatic or sympathetic peripheral or cranial nerve, except the olfactory and optic nerves, which lack Schwann's cells [3]. Approximately 45% of all schwannomas present in the head and neck region, most of them occurring in the parapharyngeal space. Benign neurogenic tumors are rare in the larynx, accounting for approximately 0.1% of all benign laryngeal tumors [5]. It has been recognized that laryngeal schwannomas arise from the internal branch of the superior laryngeal nerve after its penetration into the thyrohyoid membrane [6]. Schwannomas are usually found in 80% of the cases in the aryepiglottic folds and in the false vocal cords in the supraglottic space [7]; true vocal fold and epiglottis are more rare localization [5, 8].

Symptoms of this lesion are related to mass effect; they include hoarseness, globus sensation, sore throat, odynophagia, dysphagia, dyspnoea, stridor, and dysphonia. Symptoms progress over months to years. Stridor and dyspnoea are the late findings [7]. At least one case of death caused by acute respiratory failure has been reported [1].

Surgery is the therapy of election for this benign radioresistant tumor: they are well-circumscribed and encapsulated submucosal tumors that are extrinsic to nerve fascicles and thus are easily removed. Recurrence can follow an incomplete resection. Malignant transformation is extremely rare [5] and is observed more often in patients with VDR [9].

We describe a case of schwannoma of the aryepiglottic fold presenting with rare clinical presentation as acute respiratory distress; diagnostic findings and therapeutic measures adopted are discussed.

## 2. Case Report

A 74-year-old female was referred to Emergency Unit (EU) of our hospital for an acute dyspnoea with 1-month history of progressive globus sensation; the patient was admitted to the Pneumology Department for evaluation of an important hypercapnic respiratory failure. Arterial blood gas analysis showed pH 7.35,  *p*CO_2_ 82.9 mmHg,  *p*O_2_ 52 mmHg; chest X-ray was negative for pulmonary disease. Family history was negative for remarkable diseases. Patient's past medical history included surgical excision of a cutaneous melanoma followed by chemotherapy (presently NED), carotid thromboendoarterectomy, a surgically treated CNS meningioma with implantation of ventriculo peritoneal shunt. Traumatic lesion of vocal cords, causing mild persistent dysphonia, was reported to have occurred during previous intubation for brain surgery. In the EU, the patient underwent a flexible endoscopic evaluation of the larynx that revealed a bulky mass on the left aryepiglottic fold with the involvement of the glottic space and consequent almost complete obstruction of the laryngeal respiratory space. No palpable lymph nodes were present.

The patient was transferred to the Otolaryngology Department because of acute dyspnoea, and emergency tracheostomy was performed followed by incisional biopsy of the lesion. Intraoperatively, the mass was found to be smooth, pedunculated, and 3.5 cm in diameter. The histopathological examination of the incisional biopsy sample reported nonspecific fibrous submucosal tissue.

**Figure 1:**
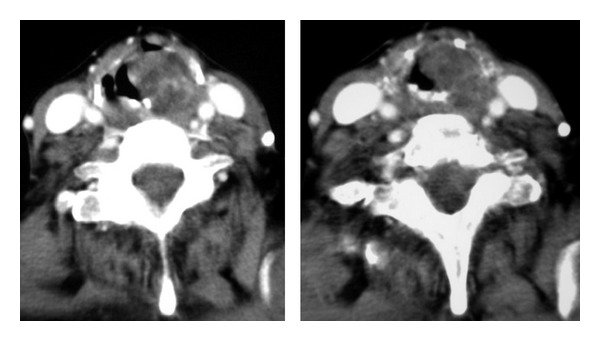
Axial CT scan showed an expansive mass occupying glottic space, originating from the left aryepiglottic fold.

CT scan demonstrated a round, heterogeneously enhancing mass, measuring 3.5 × 3 × 2.8 cm, which extended from the left aryepiglottic fold to the false vocal cord (Figure 1).

**Figure 2:**
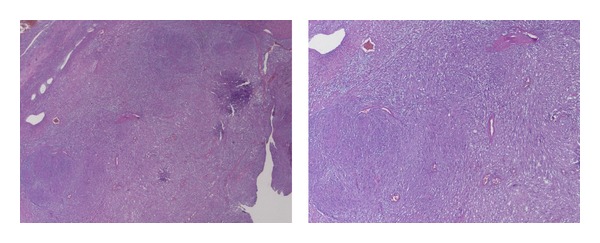
At microscopic examination, the tumor showed characteristic Antoni A and Antoni B areas.

A second single-step surgical excision was then decided. The left false vocal cord was incised with a sickle knife, and a microflap was dissected; blunt dissection revealed an encapsulated, soft tissue mass. The tumor was completely excised. Macroscopically the surgical specimen consisted of a well encapsulated tan-coloured tumor mass, measuring 4 × 2 cm. At microscopic examination, the tumor showed densely cellular areas with sheets of spindle cell palisading around amorphous matrix (Antoni A pattern) and less cellular areas with spindle cells in myxoid stroma and disorganized distribution (Antoni B pattern) (Figure 2). Tumor cells were strongly immunoreactive for S100 protein. Cellular proliferation index evaluated with Ki67 antibody was <1%.

**Figure 3:**
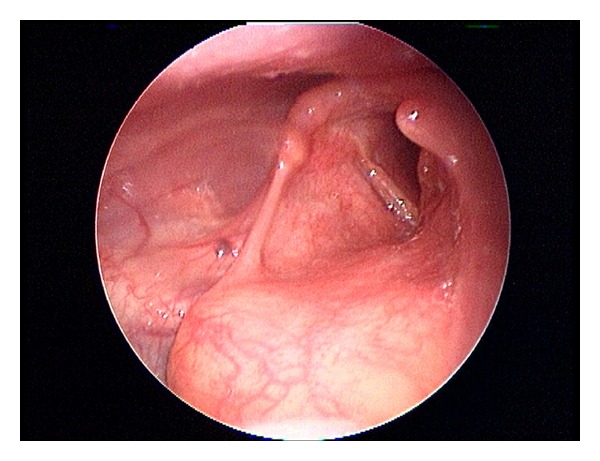
Fiberoptic laryngoscopy revealed a complete healing of the vocal cord. Laryngeal morphology and motility were preserved.

Two weeks postoperatively, the patient reported that her globus sensation, chronic throat clearing, and shortness of breath had disappeared. The tracheostomy was closed. One month after surgery, she was reevaluated in the outpatient clinic by flexible fiberoptic laryngoscopy that revealed a complete healing of the vocal cord, minimal erythema, and no edema. At 18-month followup, the patient presented no endoscopic evidence of tumor recurrence and her voice had improved markedly (Figure 3).

## 3. Discussion

In 1908, Verocay was the first to describe the tumors deriving from Schwann cells [1].

Since then, few cases have been reported in literature to date accounting for less than 0.1% of all benign tumors of the larynx [5] (Table 1).

Symptoms are related to the mass effect of a slowly growing tumour including hoarseness and foreign body sensation in the throat. As the tumor expands, it may cause dyspnoea and stridor [5]; as they are slow growing tumors, patients usually present late to the hospital and possibly acute respiratory failure in case of complete obstruction of the upper airways with a “ball and valve effects” as a result of the mass [1]. The neoplastic conditions of oropharynx and larynx more rarely present as first major symptom an acute respiratory failure by upper airway obstruction. Aspiration of a foreign body, retrosternal goiter, croup, epiglottitis occur more frequently [10]. Stridor is indicative of an upper airway obstruction, and laryngeal examination is a step in the diagnostic process [11].

Computed tomography (CT) scans recognize small schwannomas as homogeneously enhancing masses. Tumors larger than 3 cm in size are seen as masses with slightly heterogeneous contrast enhancement, suggestive of central necrosis surrounded by a peripheral enhancing ring. CT scan is used to define the extent of the lesion and its anatomic relationships [12]. The differential diagnosis includes internal laryngocele [13] and laryngeal cist [7]. At magnetic resonance imaging (MRI), T1-weighted imaging of schwannoma shows high enhancement after gadolinium injection. However, CT and MRI cannot effectively differentiate schwannomas from other benign tumours of the larynx. Definitive diagnosis of schwannoma is histological although it may be difficult to distinguish between schwannoma and neurofibroma on small samples obtained with endolaryngeal fine-needle aspiration or incisional biopsy. In the case of schwannoma, moreover, biopsy of the tumor tissue can be difficult for the presence of the capsule [12].

The treatment of choice in neurogenic tumors is a complete surgical excision that can be achieved endoscopically or through external approach depending on the localization and size of the lesion [12]. For bulky tumours tracheotomy followed by an external approach with median thyrotomy, lateral pharyngotomy or lateral thyrotomy has been described [6, 12]. Indications for the endoscopic transoral approach have been recently extended; the progress of the surgical techniques allows a direct visualization and manipulation of the lesion with a reduced risk of damaging surrounding structures during surgical procedures [14, 15]. Endoscopic excision of small lesions with successful use of CO_2_ laser has been reported with no recurrence [16]. However, the surgery must be radical, since incomplete excision may result in rapid regrowth of the tumor with life-threatening airways compromise [7]. Malignant sarcomatous changes following surgical treatment are extremely rare [10].

## Figures and Tables

**Table 1:** A review of literature of laryngeal schwannoma.

Author, year	Tumor site	Cases
Rosen et al. (2002) [7],	Aryepiglottic fold/false vocal cord	>130
Taylor et al. (2006) [5],	True vocal cord	11
Saita et al. (2005) [8],	Epiglottis	2
